# Supplementary material for: An Evaluation of Community Health Workers’ Knowledge, Attitude and Personal Lifestyle Behaviour in Non-Communicable Disease Health Promotion and Their Association with Self-Efficacy and NCD-Risk Perception
Source: Int J Environ Res Public Health. 2023 Apr 25;20(9):5642. doi: 10.3390/ijerph20095642 (PMC10178727; doi:10.3390/ijerph20095642)
Supplement: Supplementary file 1 [file ijerph-20-05642-s001.zip › ijerph-2282058-supplementary.pdf]

Information Sheet

Title of the Research Project:

Assessing and improving the competency of Urban Health Extension Workers in the prevention of non-communicable disease and promotion of healthy lifestyles in Urban districts of north Gondar Zone, northwest Ethiopia

Name of Principal Investigator (PI):

Melaku Kindie Yenit: Department of Epidemiology & Biostatistics, Institute of Public Health, College of Medicine and Health Sciences, University of Gondar

Tel: +251912696513 e-mail melaku98@gmail.com

Description of the study

The aim of this study is to determine the knowledge and attitude of Urban Health Extension Workers (HEWs) on noncommunicable diseases, risk factors, and preventive strategies; HEW participation in healthy lifestyle promotion; barriers and facilitators to engaging Urban Health Extension Workers in NCDs prevention and healthy lifestyles promotion; and to assess the impact of capacity-building training on the knowledge and lifestyle promotion skills of HEWs. The study participants' participation is entirely voluntary. This study is carried out after getting the ethical approval from University of Gondar Ethics.

Purpose of the Research Project:

The purpose of this research project is to assess and improve the competency of urban health extension workers in the prevention of non-communicable diseases (NCDs) and promotion of healthy lifestyles in urban districts of north Gondar zone, northwest Ethiopia.

What will I be asked to do?

You are invited to participate in a one-to-one interview with the data collector, during which you will be asked questions about your socio-demographics, knowledge of non-communicable diseases (NCDs), risk factors, and preventive strategies. In addition, you will be asked about your confidence in providing healthy lifestyle promotion, as well as the barriers and facilitators to lifestyle promotion. A questionnaire will be used to collect quantitative data, while your voices will be recorded in qualitative data. Your voice will be coded and entered into statistical software after it has been recorded. After the results have been analyzed, all information will be kept confidential.

Benefits

Participating in this research project may provide you with direct benefits such as information about NCDs, risk factors, and preventive strategies. Furthermore, sharing your perspectives will help to improve the planning and implementation of future community-based NCD programs. However, you will not be compensated or given any incentives to participate in this research project.

Confidentiality

Your name is not required, and codes will be used instead. The recorded voice file from the qualitative study will be deleted once the interview has been transcribed, entered into software, and is ready for analysis. This research project's information will be kept strictly confidential, and any information collected about you by this study will be stored on a password-protected computer, without any personal identifiers. Except for the principal investigator, no one will have access to the information gathered. Your information will be kept strictly confidential, and no personally identifying information will be published.

Risks or discomforts

There are no risks associated with this study. If you have any concerns about the risks or discomforts, please discuss them with the researcher.

How do I agree to participate?

Your participation is entirely voluntary, and you could tell us your decision now. If you agree to participate, please put your agreement on the consent form accompanies this information sheet. If you do not want wish to participate, you may say "No" or refuse to answer any questions, and you may leave the interview at any time without any consequences.

How will I receive feedback?

Participants will not receive direct feedback. The project summary report, on the other hand, will be distributed to the North Gondar Zone health department.

Thanks for taking the time to read this information sheet!

Informed Consent form

Dear Health Extension Workers,

We, the project members working at the University of Gondar, are developing a proposal on NCD prevention and healthy lifestyle promotion. The goal of this research is to assess and improve the competency of Health Extension Workers in the prevention of non-communicable diseases and the promotion of healthy lifestyles in the Urban districts of North Gondar Zone, northwest Ethiopia. The study findings will assist urban health extension workers in providing information on NCD prevention and healthy lifestyles promotion activities. Your name will not be written on the data collection form. You have full right to refuse to participate in the study. All information you provide in this study will be kept confidential and used solely for research purposes. The interview will last no more than one hour. Before expressing your willing to participate in this study, please read the consent form statements below.

I, hereby consent to participate in the research project titled “Assessing and improving the competency of Urban Health Extension Workers in the prevention of non-communicable disease and promotion of healthy lifestyles in Urban districts of North Gondar Zone, northwest Ethiopia.”

1. I have read the provided information.
2. Procedure details and any possible risks have been thoroughly explained to my satisfaction
3. I am aware of:
   a) I may directly benefit from participating in this research to increase my knowledge on NCDs
   b) While the results of this study will be published, I will not be identified, and individual information will be kept confidential.
   c) Whether I participate or withdraw after participating has no effect on my work.
   d) I have the right to request that the interview be terminated at any time, and I have the right to withdraw from the research at any time for any reason.
   e) While the information provided will be kept strictly confidential, no identifying information will be published.

Are you voluntary to participate in this study?

☐ **Yes** ☐ **No**

Table S1: Questionnaire

Part I: Socio-demographic characteristics of Health Extension workers

Items	Response	Remark
Your age?	__________years	
What is your educational status?	1. Level 3<br>2. Level 4<br>3. Diploma<br>4. First degree<br>5. Other (specify)……………………	
What is your marital status?	1. Single<br>2. Currently married<br>3. Divorced<br>4. Widowed<br>5. Separated	
How many years have you been working in this profession?	____________years	
Have you ever attended any training related to non-communicable diseases (NCD) in past one year?	1. Yes<br>2. No	
Is there a family member diagnosed with NCD?	1. Yes 2. No 3. Do not know	
If yes for Q.107, which family member has NCD? (More than one answer is possible)	1.Parent<br>2. Siblings<br>4. Child<br>5. Others (specify)……………………	
What type of NCD your family member had? (More than one answer is possible)	1. Hypertension<br>2. Diabetes<br>3. Cancer<br>4. Cardiac illness<br>5. Asthma<br>6. COPD<br>7. Chronic liver disease<br>8. Chronic kidney disease<br>9. Other (Specify)………………	
**Part II) Lifestyle practice of health extension workers**		
**Diet and dietary salt**		
**Questions**	**Response**	**Remark**
How often do you eat fruit?	1. Never<br>2. One to three per week<br>3. Four to six per week<br>4. Once a day<br>5. Two or more per day	
How often do you eat vegetable?	1. Never<br>2. One to three per week<br>3. Four to six per week<br>4. Once a day	

	5. Two or more per day	
How often do you eat meat?	1. Never<br>2. One to three per week<br>3. Four to six per week<br>4. Once a day<br>5. Two or more per day	
How often do you eat bread?	1. Never<br>2. One to three per week<br>3. Four to six per week<br>4. Once a day<br>5. Two or more per day	
How often do you eat rice?	1. Never<br>2. One to three per week<br>3. Four to six per week<br>4. Once a day<br>5. Two or more per day	
How many tea spoons of salt do you take per day?	1. Less than one spoon<br>2. Higher than one spoon	
How often do you add salt to your food right before as you are eating it?	1. Always<br>2. Often<br>3. Sometimes<br>4. Rarely<br>5. Never	
How often do you eat processed food high in salt? (packaged salty snacks, cheese and processed meat)	1. Always<br>2. Often<br>3. Sometimes<br>4. Rarely<br>5. Never	
On average, how often do you eat meals that were not prepared at a home? Meal includes breakfast, lunch and dinner	1. Daily or almost daily<br>2. Weekly<br>3. Monthly<br>4. Less than monthly<br>5. Never	
In general, how healthy is your overall diet?	1. Excellent<br>2. Good<br>3. Fair<br>4. Poor<br>5. Not sure/don't know	
**Physical activity**		
During the past 30 days, apart from your regular job, did you participate in any physical activity example running, jogging or brisk walking?	1.Yes<br>2. No	
What type of physical activity did you spend the most time doing during the past month?	1. Brisk walking<br>2. Running/jogging<br>3. Aerobics<br>4. Gym sessions<br>5. Others (specify)------------------	

During the last 7 days, how many days did you do vigorous physical activities like heavy lifting, digging, aerobics, or fast bicycling?	1. _____ days per week<br>2. No vigorous physical activities	
How much time did you usually spend doing vigorous physical activities on one of those days?	1. _____ hours per day<br>2. _____ minutes per day<br>3. Don't know/Not sure	
During the last 7 days, on how many days did you do moderate physical activities like carrying light loads, bicycling at a regular pace, or doubles tennis? Do not include walking.	1. _____ days per week<br>2. No moderate physical activities	
How much time did you usually spend doing moderate physical activities on one of those days?	1. _____ hours per day<br>2. _____ minutes per day<br>3. Don't know/Not sure	
During the last 7 days, on how many days did you walk for at least 10 minutes at a time?	1. _____ days per week<br>2. No walking	
During the last 7 days, how much time did you spend sitting on a week day?	1. _____ days per week<br>2. _____ minutes per day<br>3. Don't know/Not sure	
How do you perceive your weight?	1. Under weight<br>2. Normal weight<br>3. Over weight<br>4. Obese	
How would you rate your physical activity level of walking briskly, running?	1. Level1-little or no activity<br>2. Level 2-occasional activity<br>3. Level 3- regular physical activity at least 3 times per week	
Does your work primarily involve:	1. Sittting<br>2. Standing<br>3. Walking or other exercise<br>4. Heavy labour	
**Alchol drinking**		
How often do you have a drink containing alcohol?	**0.** Never<br>**1.** Monthly or less<br>**2.** 2 to 4 times a month<br>**3.** 2 to 3 times a week<br>4. 4 or more times a week	
How many drinks containing alcohol do you have on a typical day when you are drinking?	**0.** 1 or 2<br>**1.** 3 or 4<br>**2.** 5 or 6<br>3. More than 6	
How often do you have three or more drinks on one occasion?	**0.** Never<br>**1.** Less than monthly<br>**2.** Monthly<br>**3.** Weekly<br>4. Daily or almost daily	
How often during the last year have you failed to do what was normally expected from you because of drinking?	**0.** Never<br>**1.** Less than monthly<br>**2.** Monthly	

	**3.** Weekly<br>Daily or almost daily	
How often during the last year have you had a feeling of guilt or remorse after drinking?	**0.** Never<br>**1.** Less than monthly<br>**2.** Monthly<br>**3.** Weekly<br>**4.** Daily or almost daily	
How often during the last year have you been unable to remember what happened the night before because you had been drinking?	**0.** Never<br>**1.** Less than monthly<br>**2.** Monthly<br>**3.** Weekly<br>**4.** Daily or almost daily	
**Part II: General knowledge questions on NCD**		
**Prompt**	**Response**	**Remark**
Which of the following diseases are non-communicable?	A. Cancer ☐ Yes ☐ No<br>B. Hepatitis ☐ Yes ☐ No<br>C. Malaria ☐ Yes ☐ No<br>D. High blood pressure ☐ Yes ☐ No<br>E. HIV/AIDS ☐ Yes ☐ No<br>F. Cardiovascular diseases ☐ Yes ☐ No<br>G. Mental health conditions ☐ Yes ☐ No<br>H. Chronic respiratory diseases ☐ Yes ☐ No<br>I. Sickle cell anemia ☐ Yes ☐ No<br>J. Diabetes ☐ Yes ☐ No<br>K. Tuberculosis ☐ Yes ☐ No	
Can non-communicable diseases be prevented?	1. Yes<br>2. No	
Can non-communicable disease be prevented by avoiding smoking?	1. Yes<br>2. No	
Can non-communicable disease be prevented by regular exercise?	1. Yes<br>2. No	
Can non-communicable disease be prevented by proper diet?	1. Yes<br>2. No	
Can non-communicable disease be prevented by limiting alcohol consumption?	1. Yes<br>2. No	
Can non-communicable diseases be treated?	1. Yes<br>2. No	
Which one of the following is a risk factor for non-communicable diseases?<br>(**Multiple answers are possible**)	☐ Older age<br>☐ Genetic or family history<br>☐ Being overweight/obese<br>☐ Smoking<br>☐ Alcohol drinking<br>☐ Physical activity<br>☐ High salt intake	

	☐ High sugar intake<br>☐ Eating fruits and vegetables<br>☐ Stress<br>☐ Other (specify) ……………………	
Which dietary option should be taken regularly by individuals with non-communicable diseases?	☐ Foods that are high in fat<br>☐ Soft drinks and energy drinks<br>☐ High fiber foods<br>☐ Foods rich in carbohydrate<br>☐ Fruits and vegetables<br>☐ Others (specify)………………………………	
How important is for people with non-communicable diseases to eat fruit and vegetables every day?	☐ Not important<br>☐ Moderately important<br>☐ Very important<br>☐ Important<br>☐ Not sure	
A person can recognize early when they have non-communicable diseases before diagnosis?	1. Yes<br>2. No<br>3. I don't know	
**Part III) Knowledge of Specific NCDs (DM, High blood pressure, and cardiovascular disease)**		
**B1. High Blood Pressure (HBP)**		
How much do you know about high blood pressure?	☐ Nothing<br>☐ Only heard the term before<br>☐ A little about it<br>☐ Familiar with it	
If you have a family history of hypertension, you are at risk of developing high blood pressure	0. Yes 1. No 2. Not sure	
Hypertension is becoming common in Ethiopia	0. Yes 1. No 2. Not sure	
Hypertension is another name for high blood pressure	0. False 1. True 2. Not sure	
What is the abnormal level of high blood pressure?	-------- Systolic<br>------------ Diastolic	
Hypertension can be treated with medication	0. False 1. True 2. Not sure	
Lifestyle change such as weight loss can decrease blood pressure	0. False 1. True 2. Not sure	
Lifestyle change such as smoking cessation can decrease blood pressure	0. False 1. True 2. Not sure	
Damage to the kidney can be a sign of high blood pressure	0. False 1. True 2. Not sure	
Regular exercise reduces blood pressure	0. False 1. True 2. Not sure	

How much do you know about cardiovascular disease?	☐ Nothing<br>☐ Only heard the term before<br>☐ A little about it<br>☐ Familiar with it	
The older a person is, the greater his/her risk of having cardiovascular disease.	1. True<br>2. False<br>3. I don't know	
Keeping blood pressure under control will reduce risk for developing CVD	1. True<br>2. False<br>3. I don't know	
Which of the following diseases are cardiovascular (CVDs)? (**More than one answer possible)**	☐ Stroke<br>☐ Cancer<br>☐ Diabetes<br>☐ HIV/AIDS<br>☐ High blood pressure<br>☐ Malaria<br>☐ Tuberculosis	
Which of the followings are risk factors for cardiovascular diseases (CVDs)?<br>**(More than one answer possible)**	1. Having regular physical activity<br>2. High salt intake<br>3. High blood pressure<br>4. Fatty meal<br>5. High sugar intake<br>6. Avoiding smoking<br>7. Alcohol consumption<br>8. Being long-time sitting/sitting idle	
Which one of the following are preventive measures for cardiovascular diseases (CVDs)? (more than one answer possible)	1. Regular physical activity<br>2. Reduce High salt intake<br>3. Reduce Fatty meal<br>4. Reduce High sugar intake<br>5. Avoiding smoking<br>6. Avoid long-time sitting/sitting idle<br>7. All of the above	
Cardiovascular diseases (CVDs) are becoming common in Ethiopia.	0. False 1. True 2. I do not know	
How much do you know about diabetes mellitus?	☐ Nothing<br>☐ Only heard the term before<br>☐ A little about it<br>☐ Familiar with it	
Diabetes is when there is too much sugar in the blood	0. False 1. True2. I do not know	
Type 1 Diabetes is a condition of insufficient insulin production	0. False 1. True 2. I do not know	
Type 2 DM is a condition of the body which not responding for	0. False 1. True 2. I do not know	

insulin		
Type 2 DM is common among elders	0. False 1. True 2. I do not know	
Can Diabetes be prevented early?	0. Yes 1. No 2. I do not know	
Diabetes can affect vital organs	0. False 1. True 2. I do not know	
Which is the following are sign and symptoms of diabetes mellitus?<br>(**More than one possible answer possible**)	1. Weight loss<br>2. Blurred vision<br>3. Excessive hunger<br>4. Feeling tired<br>5. Excessive thirst<br>6. Frequent urination, often night<br>7. Very dry skin<br>8. High blood sugar<br>9. Slow healing of wounds	
Which of the following are complications of DM?<br>(**More than one answer possible**)	1. Loss of sensation to feet<br>2. Damage to the heart<br>3. Blindness<br>4. Damage to kidney<br>5. Damage to brain<br>6. All	
Diabetes patients should do regular exercise as the course of their treatment?	1. Yes<br>2. No<br>3. Not sure	
How often diabetes patient should do exercise?	1. Once a week for at least 30 minute<br>2. All days of the week for at least 30 minutes<br>3. Once a month for at least one hour<br>4. I do not know	
Does active smoking affect health?	1. Yes 2. No 3. I don't know	
How much people must smoke to harm health?	☐ Any smoke harms health<br>☐ At least once a wee<br>☐ Only daily smoking is harmful	
Does smoking harm lungs?	1. Yes 2. No 3. I don't know	
Does smoking harm heart?	1. Yes 2. No 3. I don't know	
Do you think smoking around others could harm their health?	2. Yes 2. No 3. I don't know	
What do you think are reasons why people smoke cigarette in your community? (**more than one answer possible)**	1. To forget their problem<br>2. To relax<br>3. A sign of modernity<br>4. Peer pressure<br>5. Stressful life events<br>6. If other (mention…………	
Does alcohol drinking affect health?	1. Yes 2. No 3. I don't know	
How much people must drink to	1) Any drink can harms health	

harm health?	2) At least once a week<br>3) Only daily drinking is harmful	
Does alcohol drinking harm lungs?	1. Yes 2. No 3. I don't know	
Does alcohol drinking harm heart?	1. Yes 2. No 3. I don't know	
How often a person should do exercise in a typical week to stay healthy? (more than one answer possible)	1. 150-300 minutes of moderate-intensity aerobic physical activity<br>2. 75-150 minutes of vigorous-intensity aerobic physical activity<br>3. 2 or more days of week with muscle strengthening activity<br>4. All	

Part IV: Attitude towards promotion of healthy lifestyles on NCD by Health Extension Worker

Questions	Strongly agree	Agree	Not sure	Disagree	strongly agree
It is part of my routine practice to advice my clients to adapt healthier lifestyles	5 ☐	4 ☐	3 ☐	2 ☐	1 ☐
I think discussing lifestyle is useful	5 ☐	4 ☐	3 ☐	2 ☐	1 ☐
Discussing lifestyle is important to improve health of my clients	5 ☐	4 ☐	3 ☐	2 ☐	1 ☐
I believe my clients expect me to discuss lifestyle	5 ☐	4 ☐	3 ☐	2 ☐	1 ☐
My lifestyle counselling advice is more effective when its linked to an individual presenting with a chronic disease	5 ☐	4 ☐	3 ☐	2 ☐	1 ☐
I do not have adequate time to provide counseling on healthy lifestyle during routine home-to-home visit	5 ☐	4 ☐	3 ☐	2 ☐	1 ☐
My clients are not receptive to receiving healthy lifestyle counselling from me	5 ☐	4 ☐	3 ☐	2 ☐	1 ☐
Discussing healthy lifestyle behaviors with my clients is very rewarding for me	5 ☐	4 ☐	3 ☐	2 ☐	1 ☐
The current structure of our health care practice limits me to engage in lifestyle counseling	5 ☐	4 ☐	3 ☐	2 ☐	1 ☐
Having more knowledge about healthy lifestyle will help my clients to improve their lifestyle practice	5 ☐	4 ☐	3 ☐	2 ☐	1 ☐

Whom do you feel is most responsible for providing healthy lifestyle promotion to people with NCD?	☐ Doctors<br>☐ Nurses<br>☐ Health officers<br>☐ Health extension workers<br>☐ All
What do you think discussing healthy lifestyle to your clients?	1. Annoying/embarrassing to clients<br>2. Easy<br>3. Difficult<br>4. Motivating<br>5. Not comfortable
What factors present a barrier to provide healthy lifestyle counseling for your clients?	1. Lack of time<br>2. Lack of knowledge<br>3. Lack of existing health care practice<br>4. Work overload<br>5. Lack of motivation<br>6. Lack of confidence of counseling<br>7. Lack of counseling supporting tool<br>8. Lack of client interest<br>9. Lack of infrastructure such as room
What do you do if your client has a too low intake of fruit and vegetables?<br>(**More than one answer possible**)	1. Ask the client for the cause of this<br>2. Provide advice to increase fruit and vegetable consumption<br>3. Provide information material (leaflet)<br>4. Refere to a dietician
Which diets do you routinely recommend?	1. Low-carbohydrate<br>2. Low-fat<br>3. low glycemic index<br>4. Very low fat<br>5. vegetarian<br>6. Dietary approaches to stop Hypertension (DASH)<br>7. I donot recommend structured specific diet in my practice<br>8. All

**Part V) NCD risk perception among Health Extension workers**					
**Domains**	**Response scale**				
**Perceived susceptibility**	Strongly agree	Agree	Uncertain	Disagree	Strongly disagree
My chances of getting NCD is great	5 ☐	4 ☐	3 ☐	2 ☐	1 ☐
Compared to others with my age and sex, I am at lower risk of NCD	5 ☐	4 ☐	3 ☐	2 ☐	1 ☐
I feel I will get NCD sometime during my life	5 ☐	4 ☐	3 ☐	2 ☐	1 ☐
I am concerned about developing cardiovascular diseases?	5 ☐	4 ☐	3 ☐	2 ☐	1 ☐
I think my personal effort will help control my risk of having NCD	5 ☐	4 ☐	3 ☐	2 ☐	1 ☐

If I had NCD, I would be depressed	5 ☐	4 ☐	3 ☐	2 ☐	1 ☐
NCD can be a serious disease if I don't prevent it.	5 ☐	4 ☐	3 ☐	2 ☐	1 ☐
If I had NCD, it would cause me to die	5 ☐	4 ☐	3 ☐	2 ☐	1 ☐
I believe maintain a normal body weight help to control NCD	5 ☐	4 ☐	3 ☐	2 ☐	1 ☐
Eating fruits can prevent me from cardiovascular disease	5 ☐	4 ☐	3 ☐	2 ☐	1 ☐
I believe that eating low sugar snacks & low-fat meals prevent NCD in the future	5 ☐	4 ☐	3 ☐	2 ☐	1 ☐
Vegetable consumption habit can prevent me from cardiovascular disease.					
Reducing dietary salt intake prevents me form cardiovascular diseases.					
I believe that regularly physical exercise will help to prevent NCD	5 ☐	4 ☐	3 ☐	2 ☐	1 ☐
Disapproval of doing physical exercise by most people nearby me could hinder me to do physical exercise	5 ☐	4 ☐	3 ☐	2 ☐	1 ☐
I do not have enough time to do physical exercise	5 ☐	4 ☐	3 ☐	2 ☐	1 ☐
High cost for fruits and vegetable could hinder me to consume those food types	5 ☐	4 ☐	3 ☐	2 ☐	1 ☐
my social network influences to drink alcohol	5 ☐	4 ☐	3 ☐	2 ☐	1 ☐
For me, stressful life events could let me to drink alcohol	5 ☐	4 ☐	3 ☐	2 ☐	1 ☐
High value given to fatty meals (meat, milk and eggs) in my community could let me to eat those foods most frequently	5 ☐	4 ☐	3 ☐	2 ☐	1 ☐
**Part VI) Perceived self-efficacy of providing health promotion on NCD**					
If it matters to my NCD health, I am confident that I can do physical exercise regularly	5 ☐	4 ☐	3 ☐	2 ☐	1 ☐
If it matters to my NCD health, I am confident that I can reduce fatty meal intake.	5 ☐	4 ☐	3 ☐	2 ☐	1 ☐
If it matters to my NCD health, I am confident that I can increase my fruit and vegetable consumption.	5 ☐	4 ☐	3 ☐	2 ☐	1 ☐
If it matters to my NCD health, I am confident that I can avoid excessive alcohol intake or not to take at all.	5 ☐	4 ☐	3 ☐	2 ☐	1 ☐

If it matters to my NCD health, I am confident that I can cease or not to take cigarette smoking.	5 ☐	4 ☐	3 ☐	2 ☐	1 ☐

Items	Strongly disagree	Disagree	Not sure	Agree	strongly agree
I am confident that I can advise my clients about prevention strategies of NCD	1 ☐	2 ☐	3 ☐	4 ☐	5 ☐
I am confident that I can advise my clients about benefits of fruits and vegetables	1 ☐	2 ☐	3 ☐	4 ☐	5 ☐
I am confident that I can advise my clients about roles of regular physical activity for NCD prevention and control	1 ☐	2 ☐	3 ☐	4 ☐	5 ☐
I am confident that I can advise my clients about the harms of cigarette smoking	1 ☐	2 ☐	3 ☐	4 ☐	5 ☐
I am confident that I can advise my clients about the harms of drinking alcohol	1 ☐	2 ☐	3 ☐	4 ☐	5 ☐
I am confident that I can advise diabetes patients about the foot care	1 ☐	2 ☐	3 ☐	4 ☐	5 ☐
I am confident that I can identify individuals at high-risk for NCDs and refer them to higher-level care	1 ☐	2 ☐	3 ☐	4 ☐	5 ☐
I am confident that I can provide guidance on medication adherence, and disease specific knowledge	1 ☐	2 ☐	3 ☐	4 ☐	5 ☐
I am confident that I can conduct capillary glucose testing-based screening	1 ☐	2 ☐	3 ☐	4 ☐	5 ☐
I am confident that I can measure blood pressure					

Supplementary Table S2: Knowledge of specific NCDs (diabetes, hypertension, and cardiovascular disease

NCDs knowledge by specific NCDs	Knowledge score		Tertile knowledge score		
			< 33th percentile	33-66th percentile	>66th percentile
	Total score	Mean (95% CI)	Low N (%)	Medium N (%)	High N (%)
**Diabetes Mellitus (DM)**					
Overall score	22	16.8 (16.4 -17.2)	56 (28.7)	49 (25.1)	90 (46.2)
General awareness	8	6.4 (6.1- 6.6)			
Signs and symptoms	9	6.7 (6.5 - 7.0)			
Complications	5	3.7 (3.5 - 3.9)			
**Hypertension**					
Overall score	10	6.4 (6.2 - 6.6)	48 (23.9)	110 (54.7)	43 (21.4)
General awareness	4	1.5 (1.4 - 1.7)			
Diagnostic criteria	2	1.6 (1.5 - 1.7)			
Management	4	3.2 (3.1- 3.4)			
**Cardiovascular disease (CVD)**					
Overall score	22	18.2 (17.8 - 18.7)	30 (15.1)	97 (49.0)	71 (35.9)
General awareness	9	7.7 (7.6 - 8.0)			
Risk factors	7	5.6 (5.4 - 5.9)			
Preventive strategy	6	4.9 (4.6 - 5.1)			
